# Neoantigens in cancer immunoediting: from mechanisms to personalized vaccines in breast cancer

**DOI:** 10.3389/fcell.2025.1733549

**Published:** 2026-01-06

**Authors:** Shiyan Zeng, Li Wang, Wanqin Zeng, Lei Sun

**Affiliations:** 1 Department of Breast, Sichuan Clinical Research Center for Cancer, Sichuan Cancer Hospital & Institute, Sichuan Cancer Center, Affiliated Cancer Hospital of University of Electronic Science and Technology of China, Chengdu, China; 2 Department of Radiation Oncology, Sichuan Clinical Research Center for Cancer, Sichuan Cancer Hospital & Institute, Sichuan Cancer Center, Affiliated Cancer Hospital of University of Electronic Science and Technology of China, Chengdu, China

**Keywords:** DNA vaccine, immunoediting, neoantigen, personalized vaccine, triple-negative breast cancer, tumor heterogeneity

## Abstract

Tumor neoantigens, a class of entirely novel antigens generated by somatic mutations, can be specifically recognized by T cells and serve as a central bridge connecting tumor genomic variation with anti-tumor immune responses. This review systematically elaborates on the dual role of neoantigens in the dynamic process of immunoediting: they act as targets for immune attack that are “sculpted” and as drivers of tumor evolution that are “selected.” It further explores their immense potential as targets for personalized immunotherapy. By delving into the mechanisms of neoantigen generation, identification strategies, and their pivotal role within the cancer-immunity cycle, the review focuses on the latest advances in neoantigen-based DNA, RNA, and synthetic peptide vaccines. Notably, drawing on a first-in-human clinical trial of a neoantigen DNA vaccine in triple-negative breast cancer (TNBC), it validates the safety, clinical feasibility, and potent immunogenicity of this therapeutic strategy. Finally, the article discusses how to address core challenges such as tumor heterogeneity and immune escape by integrating cutting-edge strategies including artificial intelligence prediction, rational multi-epitope design, and combination therapies. This provides a solid theoretical foundation and promising clinical translation prospects for personalized immunotherapy in breast cancer and other solid tumors.

## 1 Introduction

Cancer remains a major global health challenge, with breast cancer being the most common malignancy in women (Kim et al., 2025). Traditional treatment modalities, including surgery, radiotherapy, chemotherapy, and targeted therapy, often have limited efficacy due to tumor heterogeneity and the development of resistance. Even after initial successful treatment, managing recurrence—such as choosing between repeat breast-conserving surgery or mastectomy for ipsilateral breast tumor recurrence in DCIS—poses significant challenges and impacts long-term outcomes (Li et al., 2021). In recent years, immunotherapy, particularly immune checkpoint inhibitors (ICIs), has achieved breakthrough success in some cancer types (Ye et al., 2023). However, in breast cancer, except for a subset of triple-negative breast cancer (TNBC), the efficacy of ICIs as monotherapy is limited, prompting the search for novel immunotherapeutic strategies (Michaels et al., 2024).

Tumor neoantigens are aberrant polypeptides generated by tumor cells due to genomic mutations and represent a class of tumor-specific antigens (TSA) (Shang et al., 2022). These mutant peptides can be presented by MHC-I molecules and are recognized as “non-self” by T-cell receptors, thereby eliciting specific and therapeutically active T-cell responses against tumors (Yadav et al., 2014). Compared to tumor-associated antigens (TAAs), neoantigens possess high tumor specificity and a lower risk of central immune tolerance, making them ideal targets for immunotherapy (Wagner et al., 2018). However, under the strong selection pressure of the immune microenvironment, tumors evolve through immunoediting. This process drives immune evasion *via* multiple routes, including loss of heterozygosity in HLA, depletion of expressed neoantigens, or epigenetic silencing, ultimately leading to poor clinical outcomes (Rosenthal et al., 2019). A deep understanding of the interaction mechanisms between neoantigens and immunoediting is crucial for developing effective neoantigen-based vaccines.

This review will systematically elaborate on the dynamic changes of neoantigens, from their generation and identification to their role in the immunoediting process. It will focus on analyzing the latest advances in neoantigen-based personalized vaccines for breast cancer treatment and discuss future directions and prevailing challenges. While the principles of neoantigen biology and immunoediting are broadly applicable, this review distinctively examines these dynamics through the lens of breast cancer, with a focused analysis on TNBC. We aim to synthesize how the unique immune context and genomic landscape of breast cancer shape neoantigen evolution and influence the design of personalized vaccines, offering a tailored perspective that bridges general mechanisms with disease-specific application.

## 2 Mechanisms of neoantigen generation and immune recognition

### 2.1 Origin and diversity of neoantigens

Neoantigens are tumor-specific proteins derived from somatic mutations, which are recognized as “non-self” by T cells and serve as critical targets for adoptive cellular therapy (ACT) (Kim et al., 2022). Their defining characteristic is that they are expressed exclusively in tumor cells and not in normal tissues, thereby conferring high immunologic specificity and safety. Neoantigen production primarily stems from widespread genomic instability in tumors, with diverse sources including: SNVs represent the most prevalent source of neoantigens, generating a vast pool of candidate neo-peptides from which the few with true immunogenic potential must be identified (Muller et al., 2023). Such mutations are ubiquitous in tumors, particularly in cancer types with a high tumor mutational burden (TMB). Small insertions or deletions (indels) constitute a major source of neoantigens. A high tumor indel burden, often associated with high microsatellite instability, contributes significantly to tumor immunogenicity by generating novel amino acid sequences (Guo et al., 2023). Such mutations often confer strong immunogenicity due to their significant divergence from the wild-type sequence. Gene fusions, resulting from chromosomal rearrangements, produce chimeric proteins that represent an important class of tumor-specific antigens, capable of eliciting potent anti-tumor immunity even in tumors with low mutational burden (Yang et al., 2019). These neoantigens can be highly specific to certain cancer types, such as some sarcomas and leukemias. Dysregulated RNA splicing in tumors causes aberrant events like exon skipping, generating novel transcripts that give rise to a class of tumor-wide public neoantigens, offering a promising solution to intratumoral heterogeneity (Kwok et al., 2025). For instance, pathogenic isoforms of MAP4K4 and MAPK8 in glioblastoma have been shown to generate tumor-specific neoantigens. Certain oncogenic viruses integrate their genome into the host cell DNA, leading to persistent expression of viral proteins, which can be recognized as neoantigens by the immune system. The E6 and E7 oncoproteins of HPV serve as prototypical vaccine targets, as their viral origin renders them ideal “non-self” antigens. Similarly, in EBV-positive gastric cancers, viral antigens drive a potent antitumor response, highlighting the broad potential of targeting virus-derived antigens in immunotherapy (Bos et al., 2024). These include diverse modifications such as phosphorylation, acetylation, and the metabolite-induced cysteine carboxyethylation identified in this study. Such alterations can create neoantigenic epitopes that disrupt immune tolerance and drive pathogenic autoimmune responses (Zhai et al., 2023). For instance, in breast cancer, phosphorylation sites are enriched in HLA-I binding peptides, forming unique HLA signatures.

It is noteworthy that neoantigens from different sources vary in their immunogenicity. For example, neoantigens derived from driver gene mutations are more likely to be persistently expressed on the cancer cell surface and less prone to downregulation or loss, making them ideal vaccine targets. In contrast, neoantigens arising from non-functional passenger mutations may be eliminated through immunoediting.

### 2.2 Expression levels and selection criteria of neoantigens

The expression level of a neoantigen is a critical determinant of its immunogenic potential and clinical relevance. While genomic mutations provide the source of neoantigens, only those that are transcribed and translated at sufficient levels in tumor cells can give rise to peptides presented by MHC molecules and recognized by T cells.

Neoantigens are, by definition, derived from mutations absent in normal tissues, which confers tumor specificity and reduces the risk of autoimmune toxicity. However, the expression level of the mutant allele in the tumor must be high enough to generate adequate peptide-MHC complexes for T cell detection. Tumor-specific overexpression of mutant genes—often driven by copy number amplification, epigenetic activation, or transcriptional dysregulation—can enhance neoantigen presentation. In contrast, lowly expressed mutations may remain immunologically silent due to insufficient antigen density on the cell surface. In practice, RNA sequencing (RNA-seq) is used to quantify the expression of mutant alleles, typically reported in transcripts per million (TPM) or fragments per kilobase per million (FPKM). Mutations with high expression levels are generally prioritized, as they are more likely to be processed and presented. However, expression alone is not sufficient; the mutant peptide must also be efficiently processed, transported, and bound to MHC. Therefore, integrative algorithms often combine expression data with predicted MHC binding affinity, proteasomal cleavage patterns, and TAP transport efficiency to rank neoantigen candidates.

Higher neoantigen expression correlates with stronger T cell responses in preclinical and clinical studies. However, persistently high expression under immune pressure may also drive immunoediting, leading to the outgrowth of clones that downregulate or lose the antigen. Thus, while high expression is desirable for vaccine targeting, neoantigens derived from essential driver mutations—which are less likely to be lost—may offer more durable responses even at moderate expression levels.

### 2.3 Antigen processing and presentation pathways

The generation of a neoantigen does not automatically guarantee its effective recognition by the immune system. The mutant protein must undergo a complex intracellular process of antigen processing and presentation before it can be recognized by the T-cell receptor (TCR), thereby activating the adaptive immune response. This process primarily involves two classical pathways: 1) This pathway primarily presents endogenous antigens. Neoantigens are degraded in the cytoplasm by the proteasome into short peptides of 8–11 amino acids. These peptides are then selectively transported into the endoplasmic reticulum (ER) by the Transporter Associated with Antigen Processing (TAP), which functions as a molecular caliper that selects peptides primarily based on their length rather than specific sequence (Le et al., 2024). Within the ER, peptide binding to MHC I is critically supervised by a multivalent chaperone network, including calreticulin, which stabilizes the peptide-receptive state. The allosteric coupling between successful peptide loading and glycan processing ensures that only properly assembled pMHC complexes are released for transport to the cell surface *via* the Golgi apparatus, ultimately for CD8^+^ T cell scrutiny (Domnick et al., 2022). This pathway is key for activating killer T-cell responses. 2) This pathway primarily presents exogenous antigens. Neoantigens are phagocytosed by Antigen Presenting Cells (APCs) and degraded within endosomes/lysosomes into peptides of 13–25 amino acids. These peptides are loaded onto MHC Class II molecules and presented on the cell surface, leading to the activation of CD4^+^ helper T cells. This MHCII-restricted activation is essential for initiating and sustaining effective anti-tumor T cell responses (Kilian et al., 2023). CD4^+^ T cells play crucial roles in orchestrating immune responses, maintaining CD8^+^ T cell function, and establishing immunological memory.

Beyond these classical pathways, cross-presentation is a vital mechanism that links exogenous antigen uptake with MHC Class I presentation, a function predominantly carried out by specific subsets of dendritic cells. This process allows professional APCs to internalize tumor cell debris or apoptotic bodies, process the exogenous neoantigens, and present them on MHC-I molecules to prime naïve CD8^+^ T cells. This is particularly crucial for initiating *de novo* anti-tumor immune responses, as most tumor cells themselves are poor at activating naive T cells. The efficiency of cross-presentation is therefore a key determinant of success for many therapeutic cancer vaccines. The likelihood of a neoantigen being successfully presented and eliciting an immune response is influenced by a constellation of factors, including: In addition to binding affinity, the stability and half-life of the pMHC complex are crucial determinants of immunogenicity. The intrinsic instability of peptide-free MHC I complexes means that a long-lived pMHC complex provides a more sustained signal for productive T cell engagement (Moritz et al., 2019). The generation of a neoantigenic epitope is determined not only by the enzymatic preferences of the proteasome and lysosomal proteases, but also by the broader regulation of endo-lysosomal trafficking and maturation, as exemplified by the GSDMD p20 checkpoint. The generation of neoantigenic epitopes is determined not only by the enzymatic preferences of the proteasome and lysosomal proteases but also by the broader regulation of endo-lysosomal trafficking and maturation (Li et al., 2022). While the efficiency of peptide transport into the ER can be influenced by factors like specific N-terminal residues, a more fundamental source of variability lies in the existence of TAP-independent pathways. The efficacy of these alternative routes is governed by the intrinsic properties of specific HLA allotypes, such as their peptide-loading efficiency and stability, which can confer resistance to viral TAP inhibitors (Geng et al., 2018). Under the selective pressure of haploidentical transplantation or immunotherapy, tumors evolve multiple mechanisms to evade immune recognition. These include loss of heterozygosity (LOH) at the HLA gene cluster or loss of B2M expression, transcriptional downregulation of HLA class II molecules, and the upregulation of inhibitory checkpoints, collectively leading to immune escape and disease relapse (Rovatti et al., 2020).

Therefore, the transition from a genomic mutation to a clinically relevant “immunogenic neoantigen” is a stringent bottleneck. It requires not only the identification of the mutation but also the successful traversal of the entire processing and presentation pipeline, culminating in stable pMHC complex formation on the cell surface. This complexity underscores the importance of integrating prediction algorithms that go beyond mere MHC binding to model the entire antigen processing cascade for effective neoantigen vaccine design.

### 2.4 Immune recognition and T cell response

**FIGURE 1:**
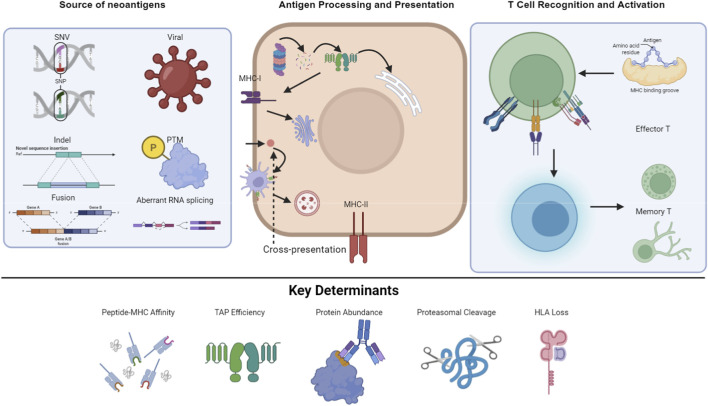
Mechanisms of neoantigen generation and immune recognition in breast cancer. Created with BioRender.com (EM294M24Z3).

After presentation by MHC molecules, neoantigens must be specifically recognized by the T-cell receptor to initiate an immune response. This process is highly specific and influenced by the diversity of the T cell repertoire. T cells recognizing neoantigens are typically not deleted during thymic development, thus possessing relatively high affinity and reactivity (Figure 1).

The integration of single-cell TCR sequencing (scTCR-seq) with transcriptomic analysis now enables more than tracking clonal dynamics; it allows for the prediction and identification of neoantigen-reactive T cell clones based on their distinct transcriptional signatures, directly from the tumor microenvironment (Lowery et al., 2022). In the phase 1 TNBC neoantigen DNA vaccine trial, immune monitoring *via* ELISpot and flow cytometry confirmed the induction of neoantigen-specific T-cell responses in 14 out of 18 patients. At a median follow-up of 36 months, the recurrence-free survival in the vaccinated cohort was a promising 87.5% (Zhang X. et al., 2024). Furthermore, the application of standardized operating procedures (SOPs) for T-cell monitoring assays, such as ELISpot and ICS, is critical to ensure data homogeneity and enable valid comparisons across different studies, as demonstrated in proficiency tests for influenza-specific immune responses (Waerlop et al., 2023).

In summary, the entire process from genomic variation to final T cell recognition of a neoantigen involves complex mechanisms at multiple levels. A deep understanding of its origin, processing, presentation, and recognition mechanisms not only aids in improving the accuracy of neoantigen prediction but also provides a theoretical foundation for optimizing vaccine design and combination therapy strategies. In the future, with the further development of artificial intelligence, single-cell multi-omics, and functional validation technologies, we can hope to more precisely screen for highly immunogenic neoantigens, advancing the clinical application of personalized cancer vaccines.

## 3 Advances in neoantigen identification technologies

The accurate identification of neoantigens is a core step in the development of personalized cancer vaccines. An ideal identification pipeline should integrate multi-omics data, bioinformatic predictions, and experimental validation to screen for targets with strong immunogenicity and broad coverage across tumor clones. Significant progress has been made in this field in recent years regarding technical methods and strategies.

### 3.1 Integrated application of multi-omics and bioinformatics

The primary step in neoantigen identification is to identify tumor-specific mutations and predict candidate peptides likely to be presented by MHC molecules and capable of activating T cells.

Whole Exome Sequencing (WES) enables the comprehensive identification of somatic mutations (SNVs and indels) by comparing tumor-normal pairs. This approach is fundamental for cross-population genomic comparisons, revealing ancestry-linked mutation patterns and clinically relevant biological insights, such as alterations in DNA damage repair pathways (White et al., 2022). This forms the fundamental basis for discovering potential neoantigenic mutations. Only expressed mutations have the potential to be processed and presented to the immune system. In the TNBC vaccine study, immunogenic neoantigens were identified using bioinformatic tools and validated in T-cell assays (Brito et al., 2023).

HLA Typing is an essential prerequisite for prediction, as neoantigen presentation is HLA-restricted. Comprehensive computational frameworks like pVACtools provide an end-to-end solution for neoantigen characterization. They integrate an ensemble of MHC Class I and II binding algorithms to predict peptide-MHC affinity, and further prioritize candidates by incorporating diverse data such as mutant allele expression and clonality, ultimately facilitating personalized vaccine design (Hundal et al., 2020). Binding affinity is typically expressed as IC50, with values below 500 nM often considered indicative of high affinity. Beyond binding affinity, an optimal candidate neoantigen should be evaluated considering multiple, refined factors: Gene and Mutant Allele Expression Level, quantified *via* RNA-seq data. Engaging multiple immune effector arms, including anti-tumor neutrophils recruited during CD4^+^ T-cell therapy, can help eliminate antigen-loss variants that arise (Hirschhorn et al., 2023). Introducing strong anchor residues (Val or Ile at P2, or Lys/Arg at the C-terminus) can enhance binding to HLA-A*1101, as these residues directly engage the MHC binding groove (Chujoh et al., 1998). This is often assessed by calculating the ratio of binding affinity between the mutant and wild-type peptides, known as the agretopicity index. According to a quantitative framework of relative immunogenicity, peptides that were not prominently presented during the establishment of deletional tolerance (such as many tumor neoantigens) retain a high degree of relative immunogenicity and are more likely to be recognized as “non-self.” The theory posits that any antigen, including an autoantigen, can attain high relative immunogenicity if presented at sufficiently high levels (van den Berg and Rand, 2004). Clinical proof-of-concept now demonstrates that neoantigens from driver mutations like KRAS G12D can be successfully targeted, yielding objective tumor regression (Leidner et al., 2022). This international consortium systematically evaluated multiple prediction algorithms by assessing 608 candidate epitopes for T cell binding. By integrating features associated with both peptide presentation and T cell recognition, the consortium built a model that filters out 98% of non-immunogenic peptides with a precision above 0.70, thereby identifying and validating the key parameters that critically influence immunogenicity (Wells et al., 2020). The integration of multi-omics data extends beyond neoantigen prediction. For example, in hepatocellular carcinoma, signatures derived from fundamental biological processes like mitotic spindle assembly have been shown to correlate with the immune microenvironment and drug susceptibility (Zhang Z. et al., 2024), underscoring the value of a holistic analytical approach in understanding tumor-immune interactions.

A variety of bioinformatic pipelines and tools have been developed to prioritize candidate neoantigens, each with distinct strengths and limitations. pVACtools offers an end-to-end workflow that integrates multiple MHC binding prediction algorithms, expression filtering, and epitope prioritization, facilitating personalized vaccine design in a user-friendly environment. However, its dependency on predefined HLA alleles and limited support for non-canonical antigen sources may constrain its utility in highly heterogeneous tumors. NetMHCpan is widely regarded as a gold standard for MHC-I binding prediction due to its high accuracy and extensive training data, yet it primarily focuses on binding affinity and does not inherently incorporate antigen processing or expression metrics. MuPeXI provides a rapid, integrated pipeline for predicting neoantigens from sequencing data, but its performance is highly contingent on the accuracy of variant calling and HLA typing. Other notable tools include NeoPredPipe, which emphasizes scalability and reproducibility, and MHCflurry, which leverages neural networks for improved affinity predictions. Despite these advances, common challenges persist across tools, including high false-positive rates, limited accuracy in predicting CD4^+^ T cell epitopes, and insufficient modeling of antigen processing and presentation dynamics. Therefore, combining multiple prediction algorithms, integrating proteomic validation data, and adopting ensemble approaches are recommended to enhance the robustness of neoantigen selection (Table 1).

**TABLE 1:** Comparison of representative computational tools for neoantigen prediction and prioritization.

Tool	Core feature	Key advantage	Main limitation
pVACtools	End-to-end workflow integrating prediction, filtering, and prioritization	User-friendly, supports MHC-I/II, facilitates vaccine design	Limited support for non-canonical antigens; relies on accurate HLA typing
NetMHCpan	High-accuracy prediction of MHC-peptide binding affinity	Considered a benchmark; broad allele coverage; continuously updated	Focuses only on binding affinity; does not integrate processing or expression data
MuPeXI	Integrated pipeline for rapid neoantigen prediction from sequencing data	Fast, streamlined, and open-source	Performance highly dependent on variant calling and HLA typing accuracy
TESLA model	Integrated model combining peptide presentation and T-cell recognition features	High precision in filtering non-immunogenic peptides; validated on large epitope sets	Model complexity; requires integration of multiple data types

### 3.2 Refinement and diversification of experimental validation techniques

Bioinformatic predictions carry a notable false-positive rate; therefore, experimental validation is an indispensable step for confirming neoantigen immunogenicity. This method involves immunoaffinity capture of naturally processed MHC-peptide complexes from cell surfaces. Following acid denaturation to dissociate the peptides, the eluted cargo is then identified using nano-ultra-performance liquid chromatography coupled to high-resolution tandem mass spectrometry (nUPLC-MS/MS), enabling the identification of thousands of peptides from diverse cell types and tissues (Purcell et al., 2019). It directly discovers peptides that are naturally processed and presented, serving as the “gold standard” for validating computational predictions. Traditional methods have low throughput and are cumbersome. Recent optimizations in cell lysis, immunoprecipitation, and peptide elution protocols have improved MS sensitivity by orders of magnitude. Combined with deep learning algorithms, the coverage and sensitivity of MHC-bound peptide identification have been significantly enhanced. Integrating MS data with genomic and transcriptomic data allows for more accurate mapping of identified peptides back to their originating somatic mutations, serving as a powerful tool for direct neoantigen discovery. These methods assess whether candidate neoantigens can be recognized by the patient’s own T cells and elicit a functional immune response. Detects IFN-γ secretion by T cells upon exposure to neoantigens, evaluating their activation. In Zhang’s clinical trial, this was one of the primary assays for monitoring immune responses (Zhang X. et al., 2024).

Building on ELISpot, ICS can further delineate whether the response is mediated by CD8^+^ or CD4^+^ T cells and provide information on T cell polyfunctionality (co-expression of multiple cytokines like IFN-γ, TNF-α), which is crucial for assessing the quality of the immune response. Utilizes fluorescently labeled pMHC complexes to directly identify and sort antigen-specific T cells *in vitro* or even from patient samples. Recent developments include encoding unique DNA barcodes into multimers, enabling high-throughput tracking of specific T cell clones. Performing TCR sequencing on tumor-infiltrating lymphocytes (TILs) or peripheral blood T cells stimulated with neoantigens can reveal the dynamic changes and expansion of vaccine-induced T cell clones. Single-cell TCR-seq (scTCR-seq) successfully identified dominant TCR clones against specific neoantigens, and their specificity was subsequently confirmed. Patient- or donor-derived T cells are co-cultured with antigen-presenting cells (dendritic cells) loaded with predicted peptides. The immunogenicity of the neoantigens is comprehensively assessed by detecting T cell activation markers (upregulation of CD137, CD69), cytokine release, or direct cytotoxic killing activity against target cells.

### 3.3 Innovative technologies and future directions

To further enhance the accuracy, efficiency, and breadth of neoantigen identification, new technologies and methods are continuously emerging.

Single-cell ECCITE-seq integrates surface protein profiling, scRNA-seq, and TCR sequencing and, together with antigen stimulation, links functional phenotypes to clonal and antigen-responsive states at single-cell resolution. This study shows that HIV-1 predominantly resides in Th1-polarized, antigen-responsive CD4^+^ T cells with high BCL2 and SERPINB9 expression; HIV-1 RNA–positive TCR clones were already expanded during viremia, persisted under virologic suppression, and were enriched for GZMB+ cytotoxic effector-memory Th1 cells, thereby directly connecting a cytotoxic CD4 phenotype, clonal persistence, and the viral reservoir (Collora et al., 2022). Developing more advanced machine learning models capable of integrating more complex datasets (epigenomic, proteomic data) and incorporating functional validation results for iterative training is key to continuously optimizing prediction algorithms. Prior to vaccine design, high-throughput *in vitro* T cell activation screening platforms can directly test the immunogenicity of large pools of candidate peptides, compensating for the limitations of purely computational predictions. Simultaneously, improved immunopeptidome enrichment techniques will aid in discovering more low-abundance but clinically relevant neoantigens. Beyond missense mutations, neoantigens can originate from aberrant RNA splicing, intron retention, dysregulated RNA editing, frameshift mutations, gene fusions, viral oncogenes, and aberrant post-translational modifications (phosphorylation, citrullination). These sources significantly expand the candidate neoantigen pool, requiring identification technologies with corresponding detection capabilities.

## 4 The dual role of neoantigens in immunoediting

**FIGURE 2:**
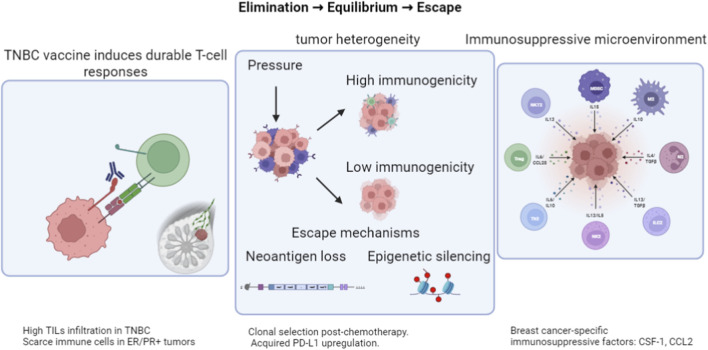
Dual role of neoantigens in cancer immunoediting dynamics. Created with BioRender.com (EM294M24Z3).

In breast cancer, the immunoediting process exhibits both universal principles and unique features shaped by subtype biology. Notably, TNBC often presents with higher tumor mutational burden and more frequent tumor-infiltrating lymphocytes, creating an initially active elimination phase. The immunogenicity and prognosis of TNBC may also be influenced by specific molecular features beyond TMB, such as the expression of genes like OBFC2A (Wu et al., 2021). Understanding these breast cancer-specific trajectories, from the clonal dynamics under chemotherapy pressure to the distinct metabolic and stromal barriers in the tumor microenvironment, is critical for timing neoantigen vaccine intervention and designing effective combination strategies. The theory of immunoediting describes a dynamic, three-phase interaction between the immune system and the tumor: elimination, equilibrium, and escape. Direct evidence from the evolution of human pancreatic cancers demonstrates that this process naturally occurs, with immune pressure selectively shaping recurrent tumors to harbor fewer high-quality neoantigens. Thus, neoantigens are key targets of immune recognition and shape tumor evolution under immune pressure *via* immunoediting (Luksza et al., 2022). A deep understanding of the mechanisms of neoantigens in each phase of immunoediting is essential for optimizing neoantigen vaccine design and improving clinical efficacy (Figure 2).

### 4.1 Elimination phase

During the elimination phase, the immune system recognizes neoantigens expressed on the surface of tumor cells, initiating a specific immune response that effectively clears transformed cells. This process involves complex mechanisms of immune recognition and activation.

The immunogenicity of a neoantigen is influenced by multiple factors. Firstly, the mutation type is a foundational factor in neoantigen generation, with sources ranging from missense substitutions and frameshift indels to viral proteins. However, effective MHC-I presentation—critical for vaccine efficacy—is not guaranteed by expression alone, as it can be hindered by intracellular processing barriers. Optimizing delivery, for instance by fusing antigens to viral carrier proteins in engineered vectors, is often essential to ensure robust immunogenic epitope presentation (Abdelaziz et al., 2019). Secondly, the degree of difference between the neoantigen and self-proteins (“non-self” character) directly impacts its immunogenicity. Greater difference results in weaker central tolerance and stronger T cell responses. Furthermore, the location of the mutation within the protein structure is important; mutations on the protein surface are more accessible for proteasomal processing and presentation. Neoantigens undergo complex intracellular processing. Intracellular proteins are degraded *via* the ubiquitin-proteasome pathway. The resulting peptides are transported into the endoplasmic reticulum by TAP, bind to MHC Class I molecules, and are presented on the cell surface, activating CD8^+^ T cells. Exogenous antigens are phagocytosed by APCs, degraded in endosomes/lysosomes, bind to MHC Class II molecules, and activate CD4^+^ T cells. In some cases, exogenous antigens can enter the MHC Class I pathway *via* cross-presentation. The activation of neoantigen-specific T cells requires a three-signal system. Signal 1 is TCR recognition of the pMHC complex; Signal 2 is co-stimulation (CD28 binding to B7); Signal 3 involves polarizing cytokines (IL-12, IFN-γ). Upon full activation, naïve T cells differentiate into effector T cells and undergo clonal expansion. In the TNBC vaccine trial, researchers detected a significant increase in IFN-γ secreting cells post-vaccination *via* ELISpot, flow cytometry showed increased proportions of IFN-γ+ cells among CD4^+^ and CD8^+^ T cells, and TCR sequencing confirmed the expansion of neoantigen-specific clones. Activated CD8^+^ cytotoxic T lymphocytes (CTLs) induce tumor cell apoptosis *via* the perforin-granzyme pathway or mediate killing through the Fas/FasL pathway. CD4^+^ Th1 cells secrete IFN-γ to activate macrophages, enhance antigen presentation, and provide cytokine support for CTL function. In some cases, CD4^+^ T cells can also directly exert cytotoxic effects. The formation of vaccine-induced immunological memory is crucial for long-term protection, as evidenced by T cell responses lasting over 12 months in the TNBC trial.

### 4.2 Equilibrium phase

The equilibrium phase is the most complex and critical stage in the immunoediting process, where the immune system, while not completely eradicating the tumor, continuously shapes tumor heterogeneity and evolutionary trajectory through selective pressure.

Under persistent immune pressure, the tumor population undergoes intense Darwinian selection. Clones expressing strongly immunogenic neoantigens are effectively eliminated by T cells, while clones with low immunogenicity or lacking neoantigens gain a selective growth advantage. This process increases tumor heterogeneity, creating an “immunoediting bottleneck.” Research indicates that neoantigens derived from driver gene mutations, although fewer in number, are less likely to be lost due to their essential role in tumor survival, making them more reliable vaccine targets. Conversely, neoantigens from passenger mutations, while more numerous, are more susceptible to elimination by selective pressure. Tumor cells employ diverse mechanisms to evade immune recognition, including gene deletion or LOH that eliminates the neoantigen source, and secondary mutations that alter the peptide sequence.Notably, the profound global loss of DNA methylation, a cancer hallmark, can paradoxically stimulate immunity by reactivating transposable elements, leading to *de novo* MHC-I presentation of TE-derived, potentially immunogenic peptides (Kong et al., 2019). In this TNBC cohort, only specific TP53 missense variants showed potential neoantigen activity, and DNA-level features (mutations/CNAs) had little prognostic value compared with RNA-based immune signatures; mechanisms underlying limited immunogenicity were not assessed (Felsheim et al., 2025). Immune selection leads to the development of complex subclonal architectures within tumors. Spatial heterogeneity enables polyclonal metastasis, evidenced by divergent genomic and immune landscapes between primary and metastatic sites. During metastasis, evolutionary selection favors Wnt wildtype subclones, which are associated with pro-metastatic, immunosuppressive microenvironments (Sun et al., 2024). Single-cell sequencing studies have moved beyond cataloging static subtypes (basal-like, mesenchymal) to reveal the dynamic forces of clonal evolution during therapy. They demonstrate that chemotherapy resistance in TNBC arises primarily through the adaptive selection of pre-existing resistant genotypes rather than through the acquisition of new mutations, while the cells undergo profound transcriptional reprogramming in response to treatment pressure (Kim C. et al., 2018).

PD-1 blockade fundamentally disrupts the equilibrium by driving a dynamic reconstitution of the T-cell repertoire. In responding tumors, this is characterized by the critical accumulation of precursor exhausted T (Texp) cells through local expansion and “clonal revival” from the periphery, rather than the reinvigoration of terminally exhausted cells (Liu et al., 2022). These changes collectively create an immunologically balanced state that permits tumor survival. To address the challenges of the equilibrium phase, next-generation vaccines employ multi-epitope strategies. To overcome tumor heterogeneity and preempt immune escape, the TNBC neoantigen DNA vaccine employed a multi-valent design, incorporating an average of 11 patient-specific neoantigens (Zhang X. et al., 2024). This strategy was intended to ensure broad immune coverage by presenting multiple non-overlapping targets. Furthermore, prioritizing neoantigens derived from clonal mutations ensures maximum coverage of the tumor cell population.

### 4.3 Escape phase

The escape phase marks the complete failure of immunological control. Tumors re-establish growth dominance through various mechanisms, ultimately leading to clinical recurrence. Understanding these mechanisms is crucial for designing effective combination therapy strategies.

Tumor cells can interfere with antigen processing and presentation pathways in multiple ways. A common immune evasion strategy involves the downregulation or loss of MHC Class I molecules, frequently achieved through B2M mutation. While this leads to resistance to CD8^+^ T cell-dependent therapies like checkpoint blockade, studies reveal that in B2M-inactivated tumors, anti-tumor immunity can be alternatively mediated by activated CD4^+^ T cells and NK cells, uncovering a compensatory immune response pathway (Torrejon et al., 2023). These alterations mean that even if neoantigens are present, they cannot be effectively presented to T cells. Regulatory T cells suppress immune responses through CTLA-4-dependent inhibition of dendritic cells (Wing et al., 2008), IL-35-mediated pathways that reinforce their suppressive function (Shao et al., 2021), and cAMP transfer to effector T cells; Myeloid-derived suppressor cells (MDSCs) deplete essential amino acids like arginine *via* arginase-1 and iNOS, producing peroxynitrites that inhibit TCR signaling; M2 macrophages secrete immunosuppressive factors like TGF-β and IL-10 to promote tolerance; Cancer-associated fibroblasts (CAFs) create physical barriers and secrete chemokines like CXCL12, limiting T cell infiltration (Song et al., 2024). Beyond stromal cells, altered signaling within the tumor cells themselves can sculpt the immunosuppressive niche. For instance, in pancreatic ductal adenocarcinoma, tumor-intrinsic Pannexin 1 channels have been shown to promote an immunosuppressive microenvironment by modulating damage-associated molecular pattern (DAMP) signaling (Wu et al., 2024), illustrating a broader mechanism of immune evasion that may be relevant across cancer types. The PD-1/PD-L1 pathway is one of the most critical immune checkpoints. Tumor cells upregulate PD-L1 expression *via* genomic amplification, PTEN loss leading to PI3K pathway activation (Zhao et al., 2020), or interferon signaling induction; other checkpoints like LAG-3, TIM-3, TIGIT are often co-expressed on exhausted T cells, synergistically inhibiting anti-tumor immunity. In TNBC, PD-L1 expression is not merely correlated with but can be directly driven by a tumor-intrinsic CD28-SNRPB2 pathway, which stabilizes Cd274 mRNA. This mechanism promotes immune escape, and its disruption can overcome anti-PD-1 resistance, suggesting that targeting tumor CD28 may effectively sensitize tumors to ICIs (Yang et al., 2025).

Beyond upregulating immune checkpoints, chemotherapeutic agents themselves can sculpt an immunosuppressive microenvironment conducive to escape. For instance, paclitaxel has been shown to promote immune dysfunction and activate the transcription factor AP-1, which in turn enhances the activity of viral promoters and drives the expression of inflammatory signaling networks such as IL-17, NF-κB, and MAPK pathways (Chen et al., 2024). This illustrates how chemotherapy can inadvertently fuel pro-tumorigenic and immunosuppressive transcriptional programs, highlighting the need for careful sequencing and combination strategies when integrating cytotoxic agents with immunotherapy.

Metabolic reprogramming in the TME profoundly impacts T cell function. Glucose competition leads to limited glycolysis in T cells, affecting IFN-γ production and cytotoxicity; While the tryptophan metabolite kynurenine (Kyn) promotes Treg differentiation *via* the aryl hydrocarbon receptor (AhR), this pathway can be effectively antagonized. The probiotic-derived metabolite indole-3-carboxylic acid (ICA) competes with Kyn for AhR binding, thereby suppressing Treg differentiation and enhancing anti-tumor immunity (Fong et al., 2023); Adenosine signaling through the A2A receptor can be harnessed to transiently inhibit the function of key drug efflux transporters like P-glycoprotein at the blood-brain barrier, thereby creating a therapeutic window for enhanced CNS drug delivery (Kim and Bynoe, 2016); lactate accumulation inhibits T cell proliferation and cytokine production. The aberrant tumor vasculature in glioblastoma, characterized by poor perfusion, actively hinders T cell infiltration and drives immunotherapy resistance. However, this barrier can be therapeutically remodeled; targeted expression of LIGHT *via* an AAV vector on brain endothelial cells has been shown to induce high endothelial venules and tertiary lymphoid structures, thereby rescuing T cell recruitment and anti-tumor immunity (Ramachandran et al., 2023); VEGF overexpression promotes angiogenesis while also acting as a potent immunosuppressive cytokine. It directly inhibits dendritic cell maturation by engaging neuropilin-1 (NRP-1), which then interacts with TLR4 to suppress downstream ERK and NF-κβ signaling, thereby blocking the upregulation of MHC and costimulatory molecules critical for T cell activation (Oussa et al., 2016); extracellular matrix remodeling increases tissue stiffness, forming physical barriers that restrict immune cell mobility. Addressing the complex mechanisms of the escape phase requires multi-pronged combination strategies. The combination of neoantigen vaccines with PD-1/PD-L1 inhibitors has shown synergy in multiple clinical trials; Microbiota-derived STING agonists activate the type I interferon response specifically in intratumoral monocytes. This IFN-I production is mechanistically critical for reprogramming the tumor microenvironment, as it regulates anti-tumorigenic macrophage polarization and facilitates NK cell-DC crosstalk (Lam et al., 2021), enhancing antigen presentation and T cell priming; chemotherapy and radiotherapy can induce immunogenic cell death, increasing neoantigen release and dendritic cell activation; targeting metabolic pathways, with IDO inhibitors, can improve the metabolic state of the TME.

### 4.4 Strategic optimization of neoantigen vaccines within immunoediting

To address the challenges across all phases of immunoediting, the design of modern neoantigen vaccines needs to comprehensively consider multiple dimensions: prediction accuracy, antigen selection, delivery platforms, and combination strategies.

Integrating multi-omics data is key to improving prediction accuracy. WES identifies coding mutations; RNA-seq confirms mutation expression; MS directly identifies MHC-bound peptides; AI algorithms like Neopepsee integrate multiple parameters, including peptide-MHC binding affinity, mutant transcript expression, and sequence similarity to known pathogenic epitopes, to predict immunogenicity with significantly improved specificity and reduced false-positive rates compared to conventional methods (Kim S. et al., 2018). *In vitro* validation employs established methods such as ELISpot for IFN-γ secretion, multicolor flow cytometry for cytokine production, and MHC multimer staining. Complementing these, emerging whole-blood cytokine release assays (IFN-γ and IP-10 ELISA) demonstrate high sensitivity and specificity in clinical settings, offering a streamlined approach to detect antigen-specific T cell responses (Ontiveros et al., 2014). Functional validation should emphasize assessing T cell polyfunctionality, not just quantity. Ideal vaccine antigens should possess the following characteristics: derived from clonal mutations to target the majority of tumor cells; originating from driver genes to reduce the risk of loss under immune selection; possessing strong HLA binding affinity and sufficient “non-self” character; highly expressed in the tumor and involved in important biological processes. Additionally, consideration should be given to including both CD4^+^ and CD8^+^ T cell epitopes to synergistically promote anti-tumor immunity. Different vaccine platforms have distinct advantages. DNA vaccines offer stability and production ease, often enhanced by electroporation. Conventional mRNA vaccines provide high expression efficiency and safety. The emerging self-amplifying RNA (saRNA) platform offers prolonged expression and heightened immunogenicity due to its self-adjuvanting nature, which activates interferon responses. However, this can inhibit translation, a challenge now being addressed by strategies such as encoding cis-acting innate immune inhibitors (MERS-CoV ORF4a) to boost protein expression and abate dose nonlinearity (Blakney et al., 2021a); peptide vaccines allow direct epitope delivery but are strongly HLA-restricted. Nanoparticle delivery systems can target dendritic cells, improving antigen uptake and presentation efficiency. The timing of vaccination is crucial. The adjuvant setting, with low tumor burden and less immunosuppression, is ideal; vaccination in the neoadjuvant setting allows assessment of *in vivo* immune responses; combination with radiotherapy can induce an *in situ* vaccination effect; sequential application with chemotherapy can eliminate immunosuppressive cells. Immune checkpoint inhibitors should be used after sufficient T cell activation to avoid premature suppression of the immune response. To counter tumor evolution, dynamic adjustment of vaccine strategy is needed. Circulating tumor DNA (ctDNA) sequencing enables the non-invasive and dynamic monitoring of clonal evolution, capturing real-time changes in the tumor mutational landscape—including the neoantigen repertoire—that occur during disease progression or under therapeutic pressure (Jamshidi et al., 2022); repeat biopsies provide TME information; TCR sequencing tracks clonal dynamics. Based on this information, “booster vaccines” targeting newly emerged clones can be designed, or antigen combinations can be adjusted to counter immune escape. Fully personalized vaccines, while precise, are costly and time-consuming. Shared neoantigens (KRAS G12D mutation (Leidner et al., 2022)) or tumor-associated antigens (telomerase, survivin) can be common in specific patient populations. A hybrid strategy combining personalized and shared antigens can maintain efficacy while improving feasibility.

Placing breast cancer within the broader landscape of cancer immunoediting reveals distinct challenges and opportunities compared to traditionally immunogenic, high TMB tumors such as melanoma or non-small cell lung cancer (NSCLC). Melanoma and NSCLC often harbor hundreds of somatic mutations, generating a rich repertoire of potential neoantigens. This high antigenic load frequently correlates with robust baseline T-cell infiltration and clinically meaningful responses to ICIs as monotherapy. Consequently, the immunoediting process in these tumors is characterized by intense, often Darwinian, selection pressure that can rapidly sculpt the neoantigen landscape, leading to well-documented mechanisms of acquired resistance like antigen loss or HLA downregulation.

In contrast, most breast cancers—except for a subset of TNBC and those with specific genomic features (BRCA deficiency)—exhibit a moderate to low TMB. The resulting neoantigen repertoire is typically more limited, which may constrain the breadth of spontaneous anti-tumor T-cell responses and partly explain the more modest efficacy of ICIs alone in breast cancer. However, this lower neoantigen burden may also translate to a less intensely edited tumor ecosystem at baseline. The immunoediting bottleneck, while still operative, might evolve over a different timescale or be more susceptible to therapeutic intervention. For instance, in TNBC, which occupies a middle ground with a relatively higher TMB and more frequent lymphocytic infiltration, the immune pressure from chemotherapy or emerging immunotherapies can induce a dynamic but tractable clonal evolution. This context makes TNBC a compelling model for neoantigen vaccination, where the goal is to amplify a focused, high-quality T-cell response against a curated set of clonal neoantigens to tip the balance from equilibrium toward elimination.

Furthermore, the tumor microenvironment (TME) differs substantially. While melanoma and NSCLC often feature “hot” but exhausted T-cell infiltrates, breast cancer TMEs, particularly in hormone receptor-positive subtypes, are frequently “cooler” and dominated by immunosuppressive stromal and myeloid cells. This difference underscores that effective neoantigen vaccines in breast cancer may require not only precise antigen selection but also strategic combinations aimed at remodeling the TME to support vaccine-primed T cells.

Understanding these comparative immunobiology and immunoediting dynamics is crucial. It informs the rationale for vaccine design, prioritizing quality and clonality over sheer quantity of neoantigens in breast cancer, and highlights why combination strategies, especially with agents that modulate the TME or reverse T-cell exhaustion, are likely indispensable for success in this disease.

## 5 Personalized neoantigen-based vaccine strategies

### 5.1 Vaccine platform technologies

**FIGURE 3:**
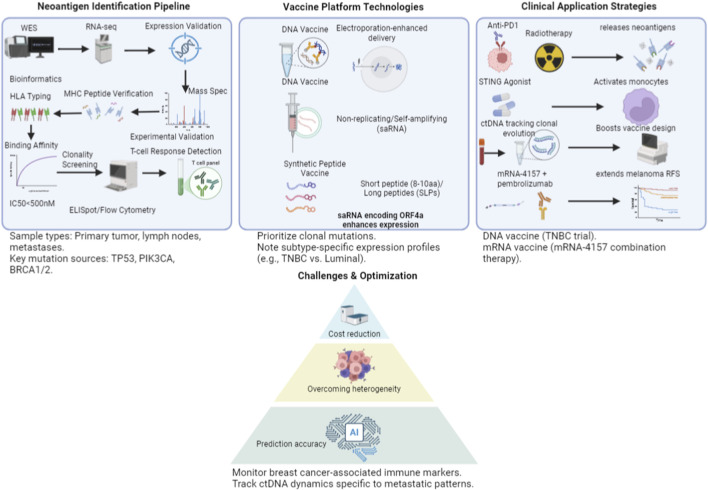
Development pipeline for personalized neoantigen vaccines in breast cancer. Created with BioRender.com (EM294M24Z3).

Personalized neoantigen-based vaccine strategies represent the cutting edge of current cancer immunotherapy. Based on the carrier and format, they are mainly categorized into three classes: DNA vaccines, RNA vaccines, and synthetic peptide vaccines. Each vaccine platform possesses distinct technical characteristics, advantages, and challenges (Figure 3).

DNA vaccines utilize plasmid DNA encoding patient-specific tumor neoantigens, delivered *via* intramuscular injection or electroporation. DNA vaccines offer advantages such as good stability, ease of production and storage, and the capacity to encode multiple epitopes. The multiantigen design of DNA vaccines leverages the host cell’s transcriptional machinery for sustained antigen expression. This facilitates the generation of broad and durable CD8^+^ and CD4^+^ T cell responses against conserved epitopes, effectively priming the immune system for accelerated responses to future viral exposures (van Bergen et al., 2023).

In recent years, DNA vaccines have shown significant progress in preclinical and clinical research. For instance, the study by Zhang et al. was the first to demonstrate the safety and immunogenicity of a neoantigen DNA vaccine in patients with TNBC(24). In a phase 1 trial of an ERBB2 ICD DNA vaccine administered intradermally with an adjuvant, the vaccine demonstrated a favorable safety profile. The majority of vaccine-related adverse events were grade 1 or 2, with no significant differences in toxicity between the three dose arms (10, 100, and 500 μg), and no severe toxicities were reported (Disis et al., 2023).

Despite their advantages, the clinical application of DNA vaccines faces challenges. Conventional naked DNA vaccines face significant challenges, including susceptibility to nuclease degradation, poor transfection efficiency, and suboptimal antigen expression. These limitations can be effectively overcome by encapsulating the plasmid DNA in cationic liposomes, which protect it from degradation and actively target it to antigen-presenting cells, thereby significantly enhancing immunogenicity (Gregoriadis et al., 2002). To overcome these limitations, the use of electroporation (EP) has emerged as a key strategy to enhance DNA vaccine delivery and immunogenicity. Future research is focused on fine-tuning EP parameters (frequency, pulse duration) to actively steer the immune response towards a Th1 or Th2 bias, enabling a tailored approach against different pathogens (Kisakov et al., 2024). Furthermore, co-delivery of the antigen-encoding plasmid with adjuvant molecules (the TLR7 agonist imiquimod) can be optimized using multifunctional nanocarriers. These systems enable synchronized delivery to the same antigen-presenting cells, where pH-responsive adjuvant release and enhanced transfection efficiency act synergistically to significantly boost vaccine immunogenicity (Lin et al., 2017).

RNA vaccines are another prominent class of personalized vaccine platforms, primarily including non-replicating mRNA vaccines and self-amplifying RNA (saRNA) vaccines. Non-replicating mRNA vaccines enhance mRNA stability and translation efficiency through optimized 5' and 3' untranslated region (UTR) structures. These vaccines encode the target antigen and are efficiently internalized by innate immune cells at the injection site; however, antigen translation primarily occurs in monocytes and dendritic cells. These antigen-presenting cells then upregulate co-stimulatory receptors (CD80/CD86), which is crucial for the subsequent priming of potent antigen-specific T cell responses (Liang et al., 2017). Self-amplifying RNA vaccines incorporate viral replication machinery (RNA-dependent RNA polymerase, RdRP), enabling intracellular RNA self-amplification and dramatically increasing antigen expression levels (Blakney et al., 2021a). These vaccines are characterized by low dose requirements and strong, durable immune responses. For instance, alphavirus vector-based self-amplifying mRNA vaccines can induce robust CD8^+^ T cell responses at lower doses, which helps establish tissue-resident memory T cells in the respiratory tract (Kunzli et al., 2022). RNA vaccines have demonstrated great potential in clinical applications. Moderna’s mRNA-4157 is a personalized neoantigen vaccine designed to target multiple patient-specific tumor antigens. By focusing on a curated set of individual-specific neoantigens, this approach aims to circumvent the potential hierarchy of immunodominance observed in shared neoantigen vaccines, and it is currently under clinical evaluation in patients with advanced or metastatic solid tumors (Rappaport et al., 2024). Building on early-phase evidence that personalized mRNA vaccines can elicit polyfunctional T-cell responses in the postsurgical setting, the combination of mRNA-4157 and pembrolizumab has received Breakthrough Therapy Designation from the US FDA. This combination is being evaluated as an adjuvant treatment for high-risk melanoma following surgery in a phase II trial (Berraondo et al., 2024). In the KEYNOTE-942/mRNA-4157-p201 trial, the combination of mRNA-4157 and pembrolizumab significantly prolonged recurrence-free survival (HR = 0.561) compared with pembrolizumab monotherapy in patients with resected high-risk melanoma (Weber et al., 2024).

However, the widespread application of RNA vaccines still faces challenges. RNA molecules are inherently unstable, requiring low-temperature storage and transportation; they may also trigger non-specific immune reactions, and the optimal delivery systems and dosing regimens are not fully defined. Beyond improving stability and delivery efficiency, the advancement of lipid nanoparticles (LNPs) has been pivotal in enhancing the immunogenicity of RNA vaccines, underpinning their successful clinical translation and regulatory approval (Blakney et al., 2021b).

Synthetic peptide vaccines directly provide immunogenic fragments of the neoantigen protein to activate specific T cell responses. Based on length, peptides are categorized as short peptides (8–10 amino acids), which typically represent single epitopes, or synthetic long peptides (SLPs, ∼20–30 amino acids), which are long enough to encompass both cytotoxic and helper T cell epitopes for a more comprehensive immune response (Kordalivand et al., 2019).

Although computationally predicted to bind directly to MHC Class I and activate CD8^+^ T cells, short peptides often exhibit limited clinical immunogenicity, a discrepancy that may stem from their susceptibility to serum degradation and the inherent limitations of prediction algorithms that overemphasize binding affinity over actual presentation (Shao et al., 2020). In contrast, SLPs offer better stability and immunogenicity. The requirement for SLP processing by antigen-presenting cells, enabling presentation *via* both MHC class I and II pathways, constitutes the fundamental mechanism for their ability to co-activate robust CD8^+^ and CD4^+^ T cell responses, which is essential for designing therapeutic vaccines that can cover a broad patient population with diverse HLA types (Jansen et al., 2023). Furthermore, SLPs can be designed to include multiple epitopes, further broadening and strengthening the immune response.

Recent research indicates that rational design of SLP structure can significantly enhance immunogenicity. For example, one study used an SLP derived from the MELOE-1 melanoma antigen containing both MHC Class I and II epitopes. The introduction of an artificial cathepsin-sensitive linker (LLSVGG) enhanced the cross-presentation of class I epitopes by dendritic cells by up to 100-fold compared to the native long peptide (Rabu et al., 2019).

Although peptide vaccines offer advantages such as high safety profile and ease of production and quality control, their clinical application has limitations. For instance, the immunogenicity of peptide vaccines is generally lower than that of DNA or RNA vaccines, and they may only provide short-term protection. Additionally, the efficacy of peptide vaccines is constrained by the patient’s HLA type, potentially leading to significant variability between individuals.

### 5.2 Clinical evidence: TNBC neoantigen DNA vaccine trial

#### 5.2.1 The pioneering TNBC neoantigen DNA vaccine trial

The groundbreaking Phase I clinical trial represented a landmark study in the neoantigen vaccine field, being the first to systematically evaluate the safety, feasibility, and immunogenicity of a personalized neoantigen DNA vaccine in patients with TNBC (Zhang X. et al., 2024). The study enrolled a cohort of high-risk TNBC patients with residual disease following neoadjuvant chemotherapy.

Researchers identified patient-specific somatic mutations through comprehensive genomic and transcriptomic profiling, then employed bioinformatic tools to predict and prioritize neoantigens with strong MHC binding affinity. Each patient received a multi-epitope DNA vaccine encoding selected immunodominant CTL, HTL, and B-cell epitopes. The epitopes were linked by short connectors and fused to an immunoenhancing adjuvant at the N-terminus *via* a specialized linker to optimize antigen processing and presentation (Pang et al., 2024).

Patients received four intramuscular injections of INO-3107 followed by electroporation. The regimen demonstrated a favorable safety profile, with treatment-related adverse events reported in 41% (13/32) of patients, all of which were low-grade. No treatment-related serious adverse events were observed (Morrow et al., 2025).

Immunogenicity evaluation through IFN-γ ELISpot and intracellular cytokine staining revealed that most patients developed neoantigen-specific T cell responses. A substantial proportion of candidate neoantigens were confirmed to be immunogenic. Epitope characterization revealed heterogeneous yet focused T cell responses, validating the high specificity of the induced immunity and successfully engaging both CD4^+^ and CD8^+^ T cell subsets (Fahnoe et al., 2025). Through T cell receptor sequencing, researchers identified and functionally validated vaccine-induced, neoantigen-specific TCR clones in multiple patients.

This pioneering work established the clinical feasibility of neoantigen vaccines in TNBC, a malignancy not traditionally considered highly immunogenic, and provided a strong rationale for their further development in breast cancer.

#### 5.2.2 Neoantigen vaccine trials in other solid tumors

The strategy of personalized neoantigen vaccination has also been actively explored in other solid tumors, demonstrating broader applicability and synergistic potential, particularly when combined with immune checkpoint blockade.

In melanoma, the combination of the personalized mRNA neoantigen vaccine mRNA-4157 with pembrolizumab has shown significant promise. In the phase 2b KEYNOTE-942 trial for resected high-risk melanoma, this combination significantly prolonged recurrence-free survival compared to pembrolizumab monotherapy (hazard ratio = 0.561), leading to its Breakthrough Therapy Designation (Weber et al., 2024). In hepatocellular carcinoma (HCC), a phase 1/2 trial evaluating a personalized neoantigen vaccine (using DNA and/or peptide platforms) combined with pembrolizumab reported an objective response rate of 30.6% in patients with advanced disease. Notably, clinical responses were correlated with the number of neoantigens encoded by the vaccine, providing a mechanistic link between vaccine design and efficacy (Yarchoan et al., 2024).

Beyond these specific cancers, early-phase trials of mRNA-4157 in combination with pembrolizumab are underway in patients with various advanced solid tumors, with preliminary evidence confirming the induction of neoantigen-specific T-cell responses (Gainor et al., 2024). In virus-associated neoplasia, the DNA vaccine platform has demonstrated utility. For instance, INO-3107, a DNA vaccine targeting HPV-6/11, showed efficacy and immunogenicity in a phase 1/2 study for recurrent respiratory papillomatosis, highlighting the versatility of the platform beyond classic somatic mutation-derived neoantigens (Mau et al., 2023) (Table 2).

**TABLE 2:** Comparison of major platforms for personalized neoantigen vaccines.

Tool	Key characteristics	Core advantage	Major challenge	Clinical stage (Example)
DNA vaccine	Plasmid DNA; delivered *via* IM injection ± electroporation	Stable, scalable production; favorable safety profile; induces both CD8^+^ and CD4^+^ T-cell responses	Requires electroporation for potent immunogenicity; theoretical risk of genomic integration	Phase I/II (TNBC)
Non-replicating mRNA vaccine	Modified mRNA; delivered *via* lipid nanoparticles (LNPs)	Rapid manufacturing; high *in vivo* expression; strong immunogenicity	Requires cold chain; potential reactogenicity; dosing optimization ongoing	Phase II/III (melanoma)
Self-amplifying RNA (saRNA) vaccine	Encodes antigen and viral replicase for intracellular RNA amplification	Potent immunity at lower doses; prolonged antigen expression	Complex manufacturing; innate immune sensing may inhibit translation	Early-phase trials
Synthetic long peptide (SLP) vaccine	Chemically synthesized peptides (20–30 aa)	Excellent safety profile; precise antigen delivery	HLA-restricted; generally lower immunogenicity compared to nucleic acid platforms	Phase I/II (various cancers)

## 6 Challenges and future directions

While neoantigen vaccines have demonstrated promising safety and immunogenicity in early trials, their broad clinical translation faces significant hurdles. Key challenges and corresponding future strategies are outlined below.

### 6.1 Enhancing neoantigen prediction accuracy

The success of neoantigen vaccines hinges on accurate target identification. Moving beyond traditional algorithms that focus solely on MHC-peptide binding affinity is crucial. AI and Multi-Omics Integration: Future efforts will integrate genomics, transcriptomics, and direct immunopeptidomics data (from mass spectrometry) with machine learning models. This holistic approach accounts for antigen expression, processing, and presentation, significantly reducing false-positive rates. High-Throughput Functional Validation: The implementation of DNA-barcoded pMHC multimers and multiplexed T cell assays is essential for experimentally validating the immunogenicity of predicted neoantigens at scale, creating a closed-loop system for model refinement.

It is noteworthy that even with advances in neoantigen prediction and validation, tumor immunoediting and heterogeneity remain significant obstacles to clinical success. For instance, the phase III trial (ACT IV) of rindopepimut, a vaccine targeting the shared neoantigen EGFRvIII in glioblastoma, failed to significantly improve overall survival (Weller et al., 2017). This study demonstrated that even well-defined, immunogenic shared neoantigens can be undermined by intratumoral heterogeneity, antigen loss, or immune escape mechanisms. These findings reinforce the necessity of multi-epitope, polyclonal coverage in vaccine design and suggest that reliance on a single shared neoantigen may be insufficient to counteract dynamic tumor evolution.

### 6.2 Overcoming tumor heterogeneity

Tumor evolution and clonal diversity are primary drivers of immune escape. This heterogeneity is often underpinned by specific oncogenic pathways that remodel the TME; for example, FGFR-related signaling signatures have been linked to immunosuppression and immunotherapy resistance in colorectal cancer (Li et al., 2024). Multi-Epitope Vaccine Design: Vaccines must target multiple clonal neoantigens derived from “truncal” mutations to preempt the outgrowth of antigen-loss variants. Dynamic Monitoring and Adaptation: Utilizing circulating tumor DNA (ctDNA) for real-time monitoring of clonal evolution will enable the development of “adaptive” booster vaccines, transforming a static treatment into a dynamic, chronic disease management strategy. This real-time adaptive approach, dynamically adjusting vaccine antigens based on ctDNA monitoring of clonal evolution, is key to anticipating immune escape. Furthermore, effective modulation of clonal evolution is achieved through the initial vaccine design by targeting multiple truncal mutations with a multi-epitope strategy, thereby reducing the outgrowth of antigen-loss variants from the outset.

### 6.3 Optimizing combination therapies

Monotherapy vaccines have limited efficacy in advanced disease; rational combinations are imperative. ICIs: Combining vaccines with PD-1/PD-L1 inhibitors can reverse T-cell exhaustion. Optimal timing—priming with the vaccine before introducing the ICI—is critical for synergy. The ability of some chemotherapies and radiotherapy to induce immunogenic cell death (ICD) provides a rationale for combining them with immunotherapy. However, translating this promising concept into clinical practice requires further validation and the identification of reliable predictive biomarkers (Rapoport and Anderson, 2019), releasing neoantigens and modulating the tumor microenvironment to enhance vaccine efficacy. Innate Immune Agonists & Targeted Therapy: Incorporating STING or TLR agonists can potentiate innate immune activation. Targeted agents (CDK4/6, PARP inhibitors) can concurrently impair tumor cells and foster a more permissive immune microenvironment.

Furthermore, the gut microbiota has emerged as a critical modulator of host response to chemotherapy and radiotherapy. Dysbiosis induced by these treatments can exacerbate intestinal damage, systemic inflammation, and immune dysfunction, whereas specific commensals and their metabolites can enhance therapeutic efficacy through metabolic reprogramming, DNA repair modulation, and immune activation, including potentiation of immune checkpoint inhibitors (Zhou et al., 2025). Therefore, microbiome-targeted interventions such as probiotics, fecal microbiota transplantation, or precision antibiotics represent a promising avenue to mitigate treatment toxicity, overcome resistance, and potentially synergize with neoantigen vaccination strategies.

### 6.4 Improving accessibility and reducing costs

The personalized nature of these vaccines presents logistical and economic challenges. Process Automation and Standardization: Streamlining and automating production—from sequencing to vaccine formulation—is key to reducing turnaround times and costs. Platform and Regulatory Innovation: Adopting cost-effective platforms (self-amplifying RNA) and developing novel regulatory pathways for “platform-based” approval of personalized products are vital steps toward democratizing access (Table 3).

**TABLE 3:** Future research priorities and roadmap.

Research direction	Key strategies	Short-term goals (1–3 Years)	Medium- to long-term goals (3–5 Years)
Prediction accuracy	Multi-omics + AI + Functional validation	Establish standardized validation platforms, prediction accuracy >80%	Achieve fully automated neoantigen screening, accuracy >95%
Overcoming heterogeneity	Multi-epitope vaccines + Dynamic monitoring	10–20 neoantigens covering major clones	Real-time adaptive vaccines, comprehensive control of clonal evolution
Combination therapy	ICIs, chemo, radiotherapy, targeted drugs, innate immune agonists	Establish 2–3 highly effective combination regimens	Personalized combination optimization, precision matching based on biomarkers
Accessibility & cost	Platform-based production, novel vectors, regulatory optimization	Preparation cycle <8 weeks, cost reduced by 50%	Preparation cycle <4 weeks, cost <100,000 RMB, insurance coverage
Research direction	Key strategies	Short-term goals (1–3 Years)	Medium- to long-term goals (3–5 Years)

### 6.5 Alternative vaccine strategies

Furthermore, while shared neoantigen vaccines (rindopepimut targeting EGFRvIII) offer advantages in standardization and lower cost, their phase III trial did not demonstrate survival benefit (Weller et al., 2017), indicating that in the context of high tumor heterogeneity, single shared antigens are vulnerable to immunoediting. Therefore, future vaccine design may consider a hybrid strategy, incorporating both personalized neoantigens and selected high-frequency shared neoantigens or tumor-associated antigens, thereby balancing personalized coverage and manufacturing feasibility.

## 7 Conclusion

Tumor neoantigens represent a paradigm shift in cancer immunotherapy, paving the way for truly personalized precision medicine. This review has elaborated on the dual role of neoantigens within the dynamic process of cancer immunoediting: they serve as targets for immune attack while also acting as a selective pressure that drives tumor evolution and immune escape. Despite the significant challenges posed by tumor heterogeneity and immunoediting, strategies employing integrated multi-omics identification technologies, advanced bioinformatic models that incorporate the entire antigen processing and presentation cascade, and rational multi-epitope vaccine design now enable the development of vaccines capable of inducing potent and polyspecific T-cell responses.

Looking forward, the broad clinical translation of neoantigen vaccines hinges on overcoming key hurdles in prediction accuracy, clonal diversity, accessibility, and cost. Addressing these challenges will require refining AI-driven prediction algorithms, developing more potent and accessible vaccine platforms, and devising rational combination therapies. As these critical technologies advance, neoantigen-based vaccines are poised to become a cornerstone of the comprehensive treatment arsenal for breast cancer. Furthermore, this therapeutic strategy holds significant promise for extension to other cancer types with low mutational burden, ultimately improving clinical outcomes for a wider spectrum of patients.
